# Quantitative Reappraisal of the Helmholtz-Guyton Resonance Theory of Frequency Tuning in the Cochlea

**DOI:** 10.1155/2011/435135

**Published:** 2011-10-19

**Authors:** Charles F. Babbs

**Affiliations:** Weldon School of Biomedical Engineering and Department of Basic Medical Sciences, Purdue University, 1246 Lynn Hall, West Lafayette, IN 47907-1246, USA

## Abstract

To explore the fundamental biomechanics of sound frequency transduction in the cochlea, a two-dimensional analytical model of the basilar membrane was constructed from first principles. Quantitative analysis showed that axial forces along the membrane are negligible, condensing the problem to a set of ordered one-dimensional models in the radial dimension, for which all parameters can be specified from experimental data. Solutions of the radial models for asymmetrical boundary conditions produce realistic deformation patterns. The resulting second-order differential equations, based on the original concepts of Helmholtz and Guyton, and including viscoelastic restoring forces, predict a frequency map and amplitudes of deflections that are consistent with classical observations. They also predict the effects of an observation hole drilled in the surrounding bone, the effects of curvature of the cochlear spiral, as well as apparent traveling waves under a variety of experimental conditions. A quantitative rendition of the classical Helmholtz-Guyton model captures the essence of cochlear mechanics and unifies the competing resonance and traveling wave theories.

## 1. Introduction

New data have created an opportunity to revisit central problem of audition: the function of the cochlea as a real-time frequency analyzer. This intellectual puzzle has attracted a large number of thinkers over the years, who have conducted extensive research in cochlear modeling [1–7]. Controversy continues, however, regarding which features of the various models are essential [8–11]. Most popular today are theories describing traveling waves that propagate longitudinally along the basilar membrane. However, criticisms of traveling wave models include suggestions that the traveling wave focusing is not sufficiently sharp and that computed peak displacements of the basilar membrane are on the order of one nanometer or less, perhaps too small to effectively stimulate hair cells [6, 12].

One path forward is to create increasingly detailed three-dimensional computational models [3, 4, 13]. The Cal Tech model of the cochlea, for example [3], uses the immersed boundary method to calculate the fluid-structure interactions at the San Diego Supercomputing Center. Six surfaces of immersed material in the cochlea are partitioned into 25 computational grids comprising 750,000 points. There is a fluid grid of 2^23^ points [3]. In one report, the simulation of two milliseconds of time required approximately 18 hours of dedicated computation on a supercomputer [4]. 

The present paper takes a much simpler approach, revisiting underlying concepts described by over a century ago by 19th Century physiologist and physicist Hermann Ludwig Ferdinand von Helmholtz [14] and elaborated in the mid-20th century by the noted physiologist, Arthur C. Guyton [15, 16]. Their classic descriptions are qualitative rather than quantitative—an omission which in the modern era is easy to remedy along the following lines.

## 2. Methods

### 2.1. The Oscillating Fluid Column Model

**Figure 1:**
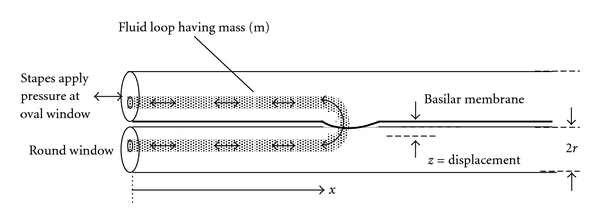
A first-principles model of an oscillating double fluid column in the cochlea. Oscillatory motion (greatly exaggerated) of a curved fluid column is shown by double arrows and by slight bulging of the resonant segment of basilar membrane. The restoring force of the basilar membrane working against the mass of fluid causes resonant behavior at a critical frequency.

Consider a mathematically idealized model of the uncoiled human cochlea in which the basilar membrane separates two long narrow compartments filled with incompressible fluid, as shown in Figure 1. Elastic membranes cover the oval and round windows at one end. The stapes act like a piston, driving very small volumes of fluid in and out at audio frequencies. The distance of travel of the stapes is exceedingly small with respect to the length of the model.

Suppose that a set of bundled, hairpin-shaped columns of incompressible fluid along the scala vestibuli and scala tympani is excited into sinusoidal, oscillatory motion in response to motion of the stapes at the oval window. Stretching of a segment of basilar membrane at the hairpin turn creates a viscoelastic restoring force and so retards motion of the fluid around the loop. Fluid oscillations occur over very small distances in one dimension along this curved path. Treatment of the parallel, one-dimensional fluid columns as mechanically independent systems is justified by spatial continuity and by the relatively low viscosity of cochlear fluid (endolymph). Variable definitions for this and related systems are listed for reference in the Nomenclature at the end of the paper.

Let *s*(*x*) be the radial span of the basilar membrane (perpendicular to the plane of the page in Figure 1) at any particular distance, *x*, from the stapes. Span *s*(*x*) is substantially less (~0.3 mm) than the diameter of either scala (~3 mm). Imagine a curved fluid column of cross section, *A*, shaded in Figure 1, extending from the oval window to the round window through a patch of basilar membrane at axial location, *x*. In this case, one can regard the fluid in the loop as a lumped mass, *m* ≈ 2*ρA*(*r* + *x*), for water density, *ρ*, and average cross section, *A*.

Suppose, as Guyton described [15], that this fluid column together with the basilar membrane constitutes a spring-mass-damper system. If the resonant frequency, f*, of this system equals the driving frequency at the stapes, then the motion of the fluid column and the corresponding local stretching of the basilar membrane are maximized because of resonance. Other parallel columns of substantially different lengths do not “feel” this resonance. Since the radius of curvature of the resonant fluid path at distance, *x*, is much greater than the distance moved by fluid particles, there is negligible angular acceleration of the fluid. Hence, fluid motion can be regarded as one dimensional. Thus, one can describe in a qualitative way a simple principle underlying place coding, in which different input frequencies result in preferential motion of the basilar membrane at different distances, *x*, from the stapes. The resonant vibrations are sensed by hair cells, connected to different 8th cranial nerve fibers, at different locations, *x*, along the membrane. In this way, the cochlea and basilar membrane could function as a real-time frequency analyzer for the nervous system.

**Figure 2:**
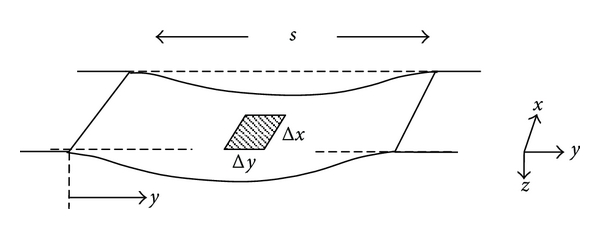
Geometry of a viscoelastic sheet stretched between parallel rigid supports. Dimensions *x* and *y* represent the axial and radial dimensions of the cochlea, respectively. The unstressed thickness of the sheet is *h*, and Young's modulus in the radial dimension is *E*. The radial span between supports is denoted *s*. A small patch of membrane of cross-section *A* = Δ*x*Δ*y* corresponds to a one-dimensional fluid column in Figure 1. The deflection at any point along the span as a function of *x*, *y*, and time, *t*, is denoted *z*. Normally *z*/*s* ≪ 1.

The goal of the present paper is to explore in a rigorous quantitative way how such a system might operate, in particular, whether a series of bundled hairpin-shaped fluid columns with graded resonant frequencies would explain the observed patterns of basilar membrane motion, including propagation of apparent traveling waves. We can begin by regarding a radial strip of basilar membrane, as shown in Figure 2, having thickness, h, as an elastic plate, subject to deformation by bending under pressure applied by the stapes to its associated fluid column. 

As shown in Appendix A, the governing equation for dynamic deformation of the elastic strip by bending (energetically much more favorable than shearing) under time varying transverse pressure *P*(*t*) is

(1a) $$\rho u\frac{\partial^{2}z}{\partial t^{2}}+\frac{1}{12}\frac{\partial^{2}}{\partial y^{2}}\left(Eh^{3}\frac{\partial^{2}z}{\partial y^{2}}\right)=P\left(t\right),$$

where *ρ* is the mass density of cochlear fluid and also of basilar membrane tissue, u is the total length of fluid along the curved path in Figure 1, *z* is local membrane deflection, *E* is Young's modulus, *h* is basilar membrane thickness, and *t* is time. For an intact cochlea *u* ≈ 2*x* + 2*r*, for mean radius, *r* of the scala tympani and scala vestibuli. 

As a biomaterial, the basilar membrane is viscoelastic. Hence, there is also damping of the oscillations by the viscosity of the basilar membrane. When viscous damping forces are included, we have

(1b) $$\begin{array}{cc} & \rho u\frac{\partial^{2}z}{\partial t^{2}}+\frac{1}{12}\frac{\partial^{2}}{\partial y^{2}}\left(Eh^{3}\frac{\partial^{2}z}{\partial y^{2}}\right)\\ & \;\;+\frac{1}{12}\frac{\partial^{2}}{\partial y^{2}}\left(Dh^{3}\frac{\partial^{2}}{\partial y^{2}}\left(\frac{\partial z}{\partial t}\right)\right)=P\left(t\right), \end{array}\;$$

where *D* is the damping modulus of the membrane material, equivalent to membrane viscosity in the radial dimension.

The corresponding expression for static equilibrium under constant pressure *P* is

(1c) $$\frac{1}{12}\frac{\partial^{2}}{\partial y^{2}}\left(Eh^{3}\frac{\partial^{2}z}{\partial y^{2}}\right)=P.$$

### 2.2. Two-Dimensional versus One-Dimensional Representations of the Basilar Membrane

**Figure 3:**
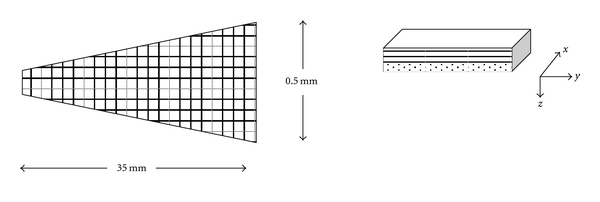
Arrangement for a two-dimensional model of the uncoiled basilar membrane including orthogonal bands of axial and radial fibers, as occurs in vivo.

In keeping with electron microscopic studies of the basilar membrane [17], it is a straightforward continuation to create a two-dimensional model that includes both radial and axial dimensions. As indicated in Figure 3, the basilar membrane is composed of layers of collagen fibers disposed at right angles. Hence, we can consider the basilar membrane as a composite in which each patch of membrane having area Δ*x*  Δ*y* is supported by layered radial and axial fibers. For this assembly, we can write

(2) $$\begin{array}{cc} & \rho u\frac{\partial^{2}z}{\partial t^{2}}+\frac{1}{12}\frac{\partial^{2}}{\partial x^{2}}\left(E_{x}h^{3}\frac{\partial^{2}z}{\partial x^{2}}\right)+\frac{1}{12}\frac{\partial^{2}}{\partial x^{2}}\left(D_{x}h^{3}\frac{\partial^{2}}{\partial x^{2}}\left(\frac{\partial z}{\partial t}\right)\right)\\ & \;+\frac{1}{12}\frac{\partial^{2}}{\partial y^{2}}\left(E_{y}h^{3}\frac{\partial^{2}z}{\partial y^{2}}\right)+\frac{1}{12}\frac{\partial^{2}}{\partial y^{2}}\left(D_{y}h^{3}\frac{\partial^{2}}{\partial y^{2}}\left(\frac{\partial z}{\partial t}\right)\right)=P\left(t\right). \end{array}$$

The spatial derivatives in the *x*-dimension reflect longitudinal coupling and describe axial forces that could lead to traveling waves. *E*_*x*_, *E*_*y*_, *D*_*x*_, and *D*_*y*_ are directional material properties.

Numerical solutions of (2) using realistic estimates for parameter values (Table 1) show that the contributions of the *x*-directed (axial) derivatives are less than one percent of the contributions of the *y*-directed (radial) derivatives. This result follows in part from the known anisotropic properties of the basilar membrane [18], the axial Young′s modulus being about 1/10th that of the radial modulus. Moreover, the curvature in the axial dimension, *d* ^2^ *z*/*dx* ^2^, is less than 1/10th that of the curvature in the radial dimension, *d* ^2^ *z*/*dy* ^2^. (Recall that the span, *s*, of the basilar membrane is ~0.3 mm, whereas the length of actively deformed basilar membrane excited by a particular tone is ~3 mm or greater.) These combined features make the directional derivatives substantially less in the axial dimension than in the radial dimension. Hence, the combined effects of stiffness and scale mean that a practical biological model can exclude axial bending forces and include only the terms in (1a)), (1b), and ((1c). (In addition to providing simplification, this insight means that “true” traveling waves are unlikely to occur, because the axial bending forces are so weak. However, apparent traveling waves can still occur, as described subsequently.) 

The mechanical problem can be simplified further by invoking the principle of condensation of degrees of freedom or “static condensation,” in which a structure is divided into substructures with the stiffness relations for each substructure generated analytically. These are combined to represent the entire structure in a condensed system of equations [19]. Static condensation can be applied to the system of Figure 1 and (1a)), (1b), and ((1c) as follows.

**Figure 4:**
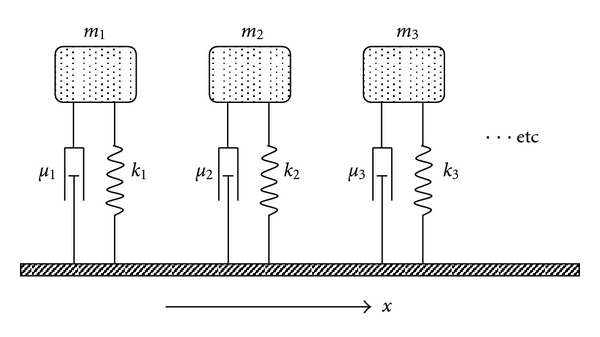
Equivalent mechanical system in one dimension, consisting of lumped masses, *m*, springs,  *k*, viscous dampers, *μ*, at various axial locations, *x*, along the basilar membrane. Parameters *k* and *μ* vary systematically as functions of *x*.

The density of collagen fibers and, hence, the stiffness of the basilar membrane vary as a function of axial position, *x*, but not as a function of radial position, *y*, as shown in electron microscopic images [17]. In the cochlear map problem, we are most interested in membrane deflection as a function of *x*. This leads to a one-dimensional Helmholtz-Guyton model of independent resonators as shown in Figure 4, in which we consider the mean deflection, $\overset{®}{z}$, across the radial span, namely $\overset{®}{z}=\left(1/s\right)\int_{\;0}^{\;s}z\left(y\right)dy$, as a function of *x* only. At each *x*-level, the basilar membrane, supported by rigid bone on one side and the spiral ligament on the other side, acts like the equivalent mechanical system in Figure 4 with effective spring, *k*, and damper, *μ*. The equivalent spring constant, *k*, is the ratio of total force *F* = *Ps*Δ*x* applied to a Δ*x* wide strip of basilar membrane with span, *s*, to the mean membrane displacement, $\overset{®}{z}$. Here *P* is the static condensation pressure in ((1c), and mean displacement, $\overset{®}{z}$, is found from integration of ((1c), as shown in Section 2.3 and in Appendix B. The mass of the cochlear fluid, *m* ≈ 2*ρ*(*r* + *x*)*s*Δ*x*, is as shown in Figures 1 and 2. For simplicity, we ignore temporarily the small additional mass, elastic forces, and damping provided by the ossicles and ossicular ligaments and also the thickness h of the basilar membrane itself (~20 micrometers). The lumped inertial mass, m, is moved by a sinusoidal force, *F*(*t*) = *P*(*t*) · *s* · Δ*x* applied by the stapes at the oval window.

In this way, a series of spring-mass-damper systems can be used to represent the average deflection of the basilar membrane, $\overset{®}{z}$, as a function of distance, *x*, from the stapes. When $\overset{®}{z}=0$, the basement membrane is in its quiet, resting state. Positive values of $\overset{®}{z}$ represent movement toward the round window. Because the stiffness, *k*, of the basilar membrane varies strongly with axial position, *x*, one can hypothesize that simple mass resonance of the various curved fluid columns between the oval and round windows is sufficient to explain the major features of cochlear mechanics. Such one-dimensional motion along a curved path, which was drawn years ago by Guyton [15], is usually interpreted today in terms of travelling waves [1, 2, 5, 20]. This paper, however, explores the alternative hypothesis that the apparent traveling waves are an epiphenomenon and that resonance of cochlear fluid along a favored path for any particular frequency constitutes the essential underlying physics.

It is straightforward to derive a suitable differential equation for any one of the lumped spring-mass-damper systems of Figure 4 and to solve it for reasonable estimates of parameters based on standard anatomy and physiology. A series of such solutions gives a predicted frequency map of the cochlea, indicating at what distances from the stapes resonance occurs for particular frequencies as well as values for the absolute magnitude of basilar membrane displacement at resonance. In Figure 4, the balance of forces at each axial location, *x*, equals mass times acceleration for a fluid column of cross section *A*(*x*) = *s*(*x*)Δ*x*, or

(3a) $$F\left(t\right)=P\left(t\right)A-kz-\mu \frac{dz}{dt}=2\rho \left(r+x\right)A\frac{d^{2}z}{dt^{2}}.$$

Rearranging (3a), the governing differential equation in terms of mean spatial displacement, *z*, of fluid around the hairpin turn as a function of time, *t*, is

(3b) $$2\rho \left(r+x\right)\frac{d^{2}z}{dt^{2}}+\frac{\mu }{A}\frac{dz}{dt}+\frac{k}{A}z=P\left(t\right),$$

where we anticipate that in general that the terms *k* and *μ*, which are constant over time, depend upon the width, thickness, and stiffness of the basilar membrane at distance, *x*, from the stapes. The mass term 2*ρ*(*r* + *x*)*A* represents a column of incompressible fluid. The assumption of an incompressible fluid is reasonable in view of the wavelength of compressive sound waves in water at audio frequencies (about 150 cm at 1 kHz), which is much longer than the dimensions of the cochlea.

Result (3b) is a relatively simple second-order differential equation which is linear in mathematical form and in underlying Newtonian physics, but which includes highly nonlinear stiffness *k* as a function of axial location, *x*. Accordingly, it is important to give special attention to the mechanical beam characteristics of a radial segment of basilar membrane, as is done in the next section.

### 2.3. Expanded Model Parameters

The next subproblem is to specify *k* and *μ* in (3a) and (3b) for a rectangular sheet of membrane suspended between a bony support and the spiral ligament having resting thickness, *h*, and Young's modulus, *E*, that crosses a gap of width, *s*, as shown in Figure 2. The condensed spring constant is equal to (pressure × area)/(mean displacement). The displacement *z*(*y*) at a particular axial position, *x*, as a function of steady pressure can be found by solving ((1c) under the boundary conditions of the problem, as shown in Appendix B. In turn, the effective spring constant, *k*, from Appendix B is

(4a) $$k=C\frac{EAh^{3}}{s^{4}},$$

so that

(4b) $$\frac{k}{A}=C\frac{Eh^{3}}{s^{4}}.$$

Here, as before, *A* = *s*Δ*x* is the area of the basilar membrane included in the local spring-mass-damper system, and *C* ~ 30 is a dimensionless numerical constant that depends upon the boundary conditions of the problem. Similarly, the effective damping factor is

(5a) $$\mu =\frac{{\text{F}}_{\mu }}{dz/dt}=C\frac{DAh^{3}}{s^{4}},$$

where *D* is the damping modulus of the membrane material, equivalent to membrane viscosity, and *F*_*μ*_ is the viscous force. In turn,

(5b) $$\frac{\mu }{A}=C\frac{Dh^{3}}{s^{4}}.$$

The constant, *C*, varies between about 25 and 60 depending on the ways that the ends of the membrane are attached and supported in the radial dimension. One possible set of boundary conditions involves “built in” supports at both inner and outer edges, for which the values of both displacement and slope are zero at both ends. In this case, the constant *C* = 60. However, along the lines of Homer et al. [21], a more realistic profile of displacement *z*(*y*) as function of radial position, *y*, consistent with experimental observations is obtained with a built in support at the inner end of the basilar membrane segment and a simple support or freely rotating end at the outer boundary. Under these conditions the constant *C* = (80/3) ≈ 26.7. (Interestingly, Bekesy (On the elasticity of the cochlear partition. J Acoust Soc Am 1948, 20 : 227–240, (1a)–(1c)) using slightly different assumptions calculated that *C* = 36 and remarked “thus the variations in width alone of the basilar membrane from cochlear apex to stapes would be enough to achieve the necessary variation in rigidity of the membrane for the analysis of frequency.”)

**Figure 5:**
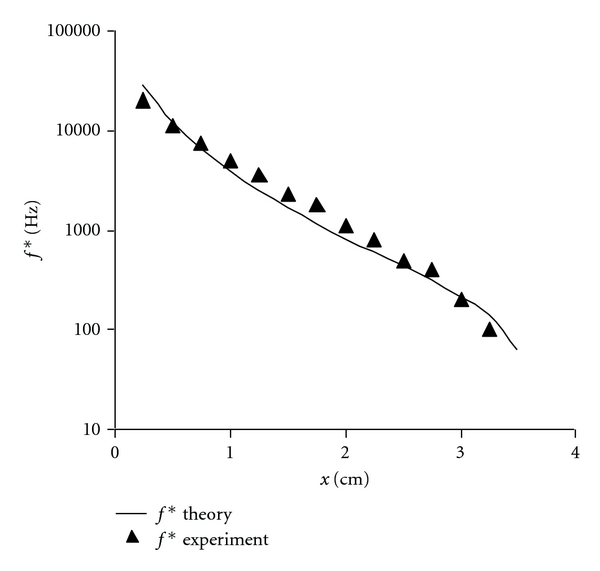
Small signal resonant frequencies for the mass resonance cochlear model as a function of distance from the stapes, compared with typical experimental data (triangles) from Greenwood [34], Figure 9. The scale is logarithmic. Stiffness constant, *C*, in expressions (4a) and (4b) and (5a) and (5b) equals 26.7 for the cochlear model.

**Figure 6:**
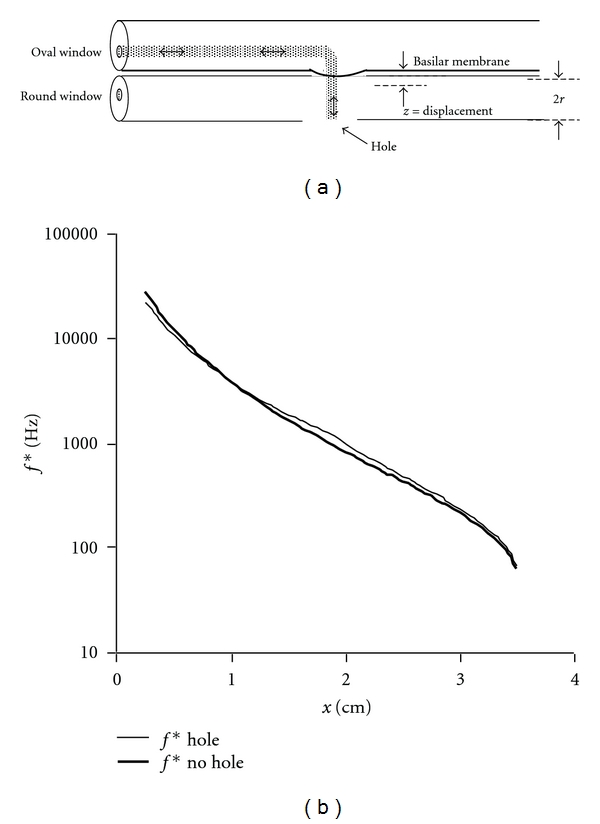
Helmholtz-Guyton model of the path for large hole drilled for observation of the basilar membrane. (a) Oscillatory motion (exaggerated, not to scale) of a curved fluid column is short circuited by the hole near the resonant segment of basilar membrane. (b) Effect of shortened path length on the frequency map of a hole at *x* = 1.67 cm.

**Figure 7:**
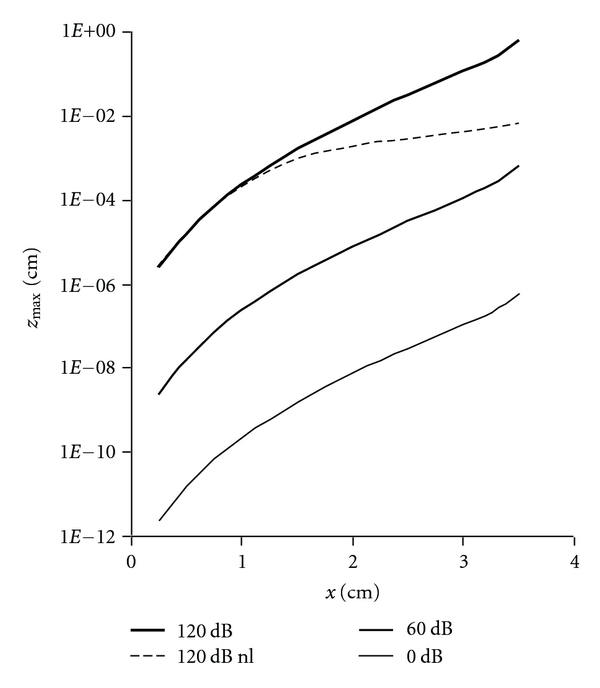
Maximal basilar membrane displacement at resonant frequency for sounds of 0, 60, and 120 dB in air as a function of distance from the stapes, computed using expression (9). The vertical scale is logarithmic. Dashed line indicates nonlinear behavior associated with membrane stretching as well as membrane bending at extreme incident sound pressure levels.

**Figure 8:**
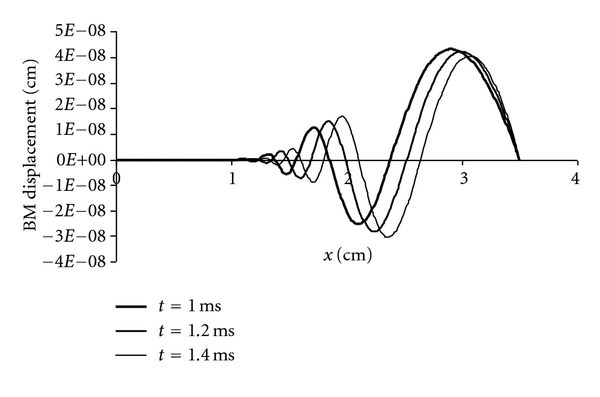
Apparent traveling waves following a transient, click-like disturbance (10) in a system of independent oscillators mimicking the basilar membrane. To mimic the effect of the helicotrema, the transient pressure across the membrane is scaled by the exponential function 1 − e^−(*x*_max⁡_−*x*)/*ζ*^ with length constant, *ζ*, equal to 1.0 cm. BM indicates basilar membrane.

**Figure 9:**
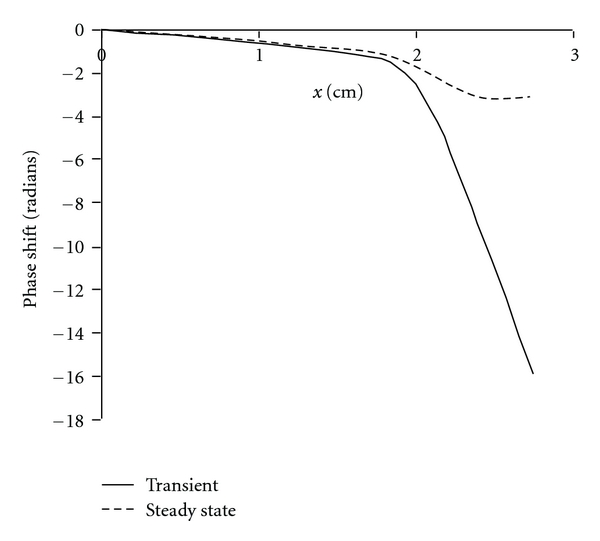
Apparent phase shifts following onset of a 1 kHz tone in a system of independent oscillators mimicking the basilar membrane. Solid curve indicates transient responses to a click. Dashed curve indicates responses to a pure continuous tone of 1 kHz.

**Figure 10:**
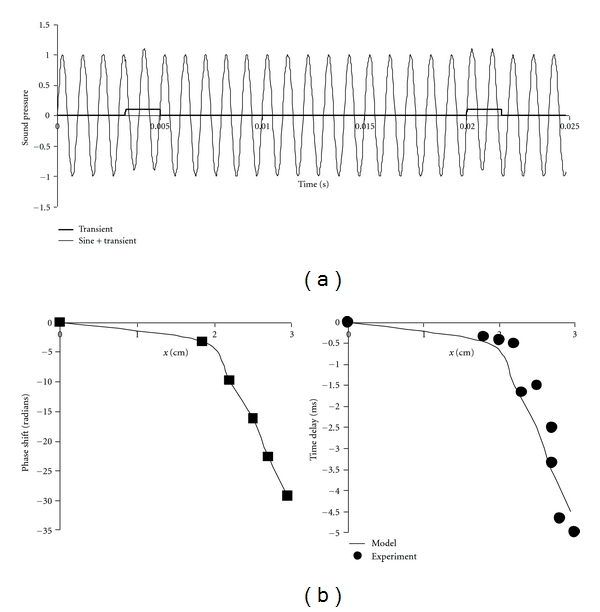
A computational thought experiment recreating von Bekesy's stroboscopic measurements of phase delays of putative traveling waves along the basilar membrane. (a) Periodic impulses (small rectangular pulses at 60 Hz) added to a continuous 1 kHz tone applied to an array of independent spring-mass-damper resonators mimicking the basilar membrane. (b) Left: apparent phase shifts with the spring-mass-damper system of Figure 4, determined by simulating von Bekesy's stroboscopic method [22, 37]. (b) Right: absolute time delays in milliseconds from model simulations at left, compared with Bekesy's experimental data from Figure  5 in reference [37]. Frequency independent absolute time delays were calculated as the phase delay in radians divided by (2*π*·frequency).

As shown in Appendix B, the parameter, *C*, can also be used to describe the effects of radial geometry of the cochlear spiral. For a curved basilar membrane with inner radius r_0_ and outer radius *r*_0_ + *s* and with collagen fibers disposed along the radial dimension from inner wall to outer wall, the same dependence of effective spring and damping constants directly on *E* and *h* ^3^ and inversely on *s^4^* obtains as in expressions (4a)–(5b). However, the parameter *C* may vary slightly with curvature. For the symmetrical boundary conditions of built-in edges at both inner and outer edges of the basilar membrane, *C* = 60 as *r*_0_ approaches zero, and *C* = 60 also as *r*_0_ becomes infinitely large. However, for the more anatomically realistic asymmetrical boundary conditions of a built-in inner edge and a pivoting outer edge, *C* = 24 as *r*_0_ approaches zero, and *C* = 26.7 as *r*_0_ becomes large (Appendix B). For realistic curvatures of the cochlea (Bekesy Experiments in Hearing [22], Figures 3–10), a value of *C* between 26 and 26.7 obtains for all distances, *x*, from the stapes. Accordingly, the uncurved or uncoiled model for the cochlea can be considered valid. The changes in *k* and *μ* associated with anchoring boundary conditions or curvature remain small, however, compared to changes associated with the fourth power of the basilar membrane span and the decreasing elastic modulus, *E*, from base to apex of the cochlea.

### 2.4. Basilar Membrane Motion at Resonance

Introducing the membrane specific spring and damping factors back into the balance of forces (1a)), (1b), and ((1c), the spatial average displacement, *z*, of the resonant segment of basilar membrane can be described by the second-order differential equation as a function of time, *t*,

(6a) $$2\rho \left(r+x\right)\ddot{z}+C\frac{Dh^{3}}{s^{4}}\dot{z}+C\frac{Eh^{3}}{s^{4}}z=P_{\max}\sin\left(\omega \text{t}\right).$$

Here, the “dot” symbol over *z* indicates the first time derivative, and the “double dot” symbol over *z* indicates the second time derivative. The angular frequency, *ω*, is the driving frequency of amplified sound pressure entering the cochlea at the oval window. For any given point along the axis of the cochlea, (6a) can be represented as

(6b) $$U\ddot{z}+V\dot{z}+Wz={\text{P}}_{\max}\sin\left(\omega \text{t}\right),$$

including lumped constants *U*, *V*, *W*, and *P*_max⁡_. Lumped constant *U* represents the effective mass of the double fluid column at a particular distance, *x*, from the stapes. Lumped constant *V* represents damping. Lumped constant *W* represents elasticity. There is separate set of constants, constants *U*, *V*, and *W* in (6b), for each axial location, *x*. In turn, there is a separate resonant frequency for each *x*. Solution of (6b) for the conditions of resonance leads to a predicted frequency map of spatial mean displacement, *z*, as a function of *x*.

The steady-state solution of (6b) is given, as shown in Appendix C, by the expression

(7) $$z_{1}=\frac{P_{\max}}{\sqrt{\left(U\omega^{2}-W^{2}\right)+V^{2}\omega^{2}}}\sin\left(\omega t+\beta \right)$$

for phase angle *β*. At steady state (tones longer than about 50 msec), the maximal positive basilar membrane deflection, *z*_max⁡_, happens when *sin*(*ωt* + *β*) = 1. Resonance occurs at angular frequency, ω*, when *U*(ω*)^2^ − *W* = 0 or

(8) $$\omega^{*}=\sqrt{\left(\frac{W}{U}\right)}.$$

Since *W* and *U* are functions of position along the basilar membrane, expression (8) specifies the frequency map. At any particular distance from the stapes, the maximal positive membrane excursion toward the round window is given by

(9) $$z_{\max}=\frac{P_{\max}}{V\omega^{*}}$$

for the conditions for resonance.

A complete solution of (6a) and (6b), however, includes both homogenous and particular solutions. Solution of the homogenous equation $U\ddot{z}+V\dot{z}+Wz=0$ can always be added to the particular solution and describes transient disturbances or “impulse responses” that may occur during onset of continuous tones or in response to clicks or impulses. The solution to the homogenous equation is

(10) $$z_{2}=\;\frac{P_{i}\Delta t}{2\rho \left(x+r\right)\lambda }\sin\left(\lambda t\right){\text{e}}^{-bt},$$

where *P*_*i*_ is the time-averaged pressure of a transient impulse, delivered over short duration Δ*t* that sets the membrane in motion, and

(11) $$\begin{array}{cc} \lambda & =\sqrt{\frac{CEh^{3}}{2\left(x+r\right)s^{4}}},\;\;b=\frac{CDh^{3}}{4\left(x+r\right)s^{4}}. \end{array}$$

The complete solution for mean basilar membrane displacement includes the sum of the transient and steady-state solutions: *z* = *z*_1_ + *z*_2_.

### 2.5. Estimates of Parameters

An important reason for revisiting the resonance theory of frequency coding in the cochlea is the availability of new anatomical and biomechanical data that allow for exact quantitative modeling. Table 1 presents estimates for model parameters obtained from literature sources indicated in the right hand column. Estimates for middle ear components for damping and elasticity (not shown) are relatively small for normal ears and are ignored in the present analysis. The anatomic scale of the final model represents a human cochlea. Some remaining variables are estimated from available animal data. Detailed comparison of species-specific models based on (6a) and (6b) is certainly possible, but beyond the scope of the present paper.

## 3. Results

### 3.1. Frequency Map of the Cochlea

Figure 5 shows a plot of resonant natural frequencies $\begin{array}{cc} f^{*} & =\omega /(2\pi ) \end{array}$ from expression (8) along the basilar membrane for the human scale mass resonance model of expression (6a) and (6b). This plot represents the frequency map of the cochlea. The predicted frequency map (smooth curve) is in reasonable agreement with the experimental results [34], as shown by the solid triangles.

The present analysis also offers a direct and simple answer to a persistent question regarding experimental work on cochlear mechanics, namely, the effect of the hole drilled in the cochlear shell to allow observations of basilar membrane movement [6, 12, 22, 35]. The first law of instrumentation, as stated by Geddes and Baker [36], is that the process of making a measurement should not interfere with the phenomenon being observed. Yet to observe and measure the amplitude of basilar membrane motions and in turn the frequency map experimentally, it is necessary to drill a hole in the surrounding bone. This is done in the scala tympani near the particular turn in the spiral cochlea where large amplitude vibrations are expected for a given frequency [12, 22]. The presence of a hole would tend to decompress the scala tympani at the point of observation and might alter pressures and flows on one side of the cochlear partition. 

In the present analysis, the effect of a large hole is easily modeled in terms of shortening the overall path length in the region of the membrane under study from approximately 2(*x* + *r*) to approximately (*x* + 3*r*), as shown in Figure 6(a). In the idealized case of a large hole that completely vents the cochlea near the characteristic place for maximal vibrations, there is a minor violation of the first law of instrumentation owing to reduced mass of the resonant fluid column. The resulting frequency map shows slight upward shift of the characteristic frequencies owing to reduced fluid column mass. However, the predominant effect of the fourth power of basilar membrane span persists.

### 3.2. Amplitude of Basilar Membrane Motion

Figure 7 shows corresponding values of *z*_max⁡_ at natural frequencies f* along the axis of the basilar membrane for 0 dB, 60 dB, and 120 dB continuous tones (Expression (9)). The absolute value of oscillatory motion predicted by the mass resonance model is noteworthy. 60 dB sounds characteristic of normal speech range from about 1 to 1000 nanometers. These theoretical findings are consistent with experimental results. For example, Rhode (Figure  7 in [12]) found 70 dB sounds produced membrane excursions of 1 to 1000 nanometers. For loud 120 dB sounds, the calculated maximal basilar membrane excursions were 1000-fold greater than for 60 dB sounds at any particular frequency, as calculated from the simple linear model of expression (9) that is based upon bending deformation only. However, when both bending and stretching forces were accounted (dashed line, methods not detailed here), nonlinear behavior emerged which limited basilar membrane excursion to less than 100 microns. Nonlinear behavior emerged only under conditions when the peak membrane excursion was greater than the thickness of the basilar membrane. For all but these loudest sounds at lower frequencies (<3 kHz), the stretching forces were negligible and a simple bending model sufficed. There is reasonable agreement between the quantitative Helmholtz-Guyton theory and experiment.

### 3.3. Traveling Waves and Phase

Historically, observations consistent with traveling waves [8, 10, 22] have been cited as the predominant reason for discounting the resonance theory of place coding in the cochlea as overly simplistic. However, the quantitative evaluation of resonance in an ordered set of spring-mass-damper systems with varying stiffness shows that apparent traveling waves can arise as an emergent property of the system under certain conditions. 

The traveling waves in Figure 8 were produced from the homogenous solution (10) following a 1 msec duration pressure impulse of 1 dyne/cm^2^. Young's modulus *E* as a function of distance, *x*, from the stapes was 2 × 10^9^((1 − *x*)/3.6) dyne/cm^2^. Damping was increased from 1000 to 4000 dyne-sec/cm^2^ to more clearly separate the waves for plotting. To mimic the effect of the helicotrema in venting transmembrane pressure differences near the apex of the cochlea, the waves are scaled by the exponential function *ϕ*(*x*) = 1 − e^−(*x*_max⁡_−*x*)/*ζ*^ with length constant, *ζ*, equal to 1.0 cm. The function *ϕ*(*x*) sets a quiet boundary condition at the apex without changing the frequency map. Each of the oscillators in an array is disposed along the basilar membrane from 0 to 3.5 cm from the stapes stimulated by pressure *P*_max⁡_ *ϕ*(*x*) at time zero. No physical connection between the oscillators was modeled to allow for conventional wave propagation. Nonetheless, apparent “traveling waves” arise. 

The same effect also produces phase relationships similar to those observed experimentally. Figure 9 illustrates apparent phase shifts computed from numerical integration (simple Euler method with time step of 1 microsecond to ensure accuracy) of (6a) and (6b) describing a one-dimensional Helmholtz-Guyton model of the basilar membrane. The characteristic place of resonance in this model is near 1.67 cm. The apparent traveling waves were observed during the first 4 msec after onset of a continuous 1 kHz tone. There is progressive delay in the time of arrival of the early peaks of sine waves at positions distal to the characteristic place, leading to the appearance of traveling waves with a progressive shift in phase calculated on the basis of the period of the exciting pulse (1 kHz). A very similar curve was presented in von Bekesy's classical paper [37] as key evidence for the existence of traveling waves. 

If the numerical simulation is continued for 100 msec or more, the phase relationships indicated by the dashed line in Figure 9 are obtained. The phase shifts from numerical solutions approach those expected from analytical solutions for steady state, ranging from zero to −*π* radians. Thus, the quantitative response of the Helmholtz-Guyton resonators to clicks gives evidence consistent with traveling waves. The response to a pure continuous tone does not.

However, larger negative phase shifts similar to those for responses to clicks can be expected for continuous tones under realistic experimental conditions, if the continuous tones contain embedded transients that stimulate repeated impulse responses.  Figure 10(a) shows a sample of such a signal in which the amplitude of the impulse is only 1/20th that of the continuous sinusoidal signal and occurs every 17 cycles. It is instructive to reconstruct Bekesy's historic experiments that seemingly rule out the resonance theory and support the traveling wave theory using a continuous sinusoidal input contaminated with a small amount of noise. In Bekesy's stroboscopic experiments [37], the cochlea was excited by a continuous tone at the stapes and a strobe flash at twice the driving frequency was used to illuminate the basilar membrane at different axial positions. The delay of the strobe was adjusted until the apparent membrane deflection at a given axial position reached a null or minimum amount of movement. This null was interpreted as indicating that the traveling wave had been delayed by a multiple of the half-cycle time.

In the present simulated reconstruction Bekesy's experiment, the basilar membrane motion was computed from numerical integration of (6a) and (6b) describing a one-dimensional Helmholtz-Guyton model of the basilar membrane. Periodic impulses were added to the sinusoidal driving pressure, as shown in Figure 10(a). Print-outs of the *z*-axis positions of the basilar membrane were made at times corresponding to the strobe flash in Bekesy's experiment and null points identified by least-squares analysis. From the location of these successive null points, the delays in arrival of the apparent traveling waves at particular locations, *x*, along the basilar membrane were computed under steady state conditions (>150 msec after onset of the continuous tone). Then the corresponding phase delays were computed, based on the driving frequency at the stapes. 

When the combined 1000 Hz tone plus 60 Hz interference was applied to the cochlear model of (6a) and (6b), apparent traveling waves persisted more than 50 msec after onset of the exciting tone and did not diminish in size. In the axial dimension, these waves traveled both toward the stapes and toward the helicotrema, propagating outward from the point of maximal deflection, with much greater amplitude in the direction of the helicotrema.

The left-hand plot in Figure 10(b) shows phase results from this computational thought experiment. Addition of small transient signals to continuous tones can result in apparent phase shifts characteristic of steady-state traveling waves in this system of physically independent resonators having graded spring constants corresponding to those of radial strips of basilar membrane. These phase shifts are similar to those observed by von Bekesy in human anatomic specimens (Figure  5 in [37]) at frequencies of 200 and 300 Hz. Absolute time delays in the arrival of apparent traveling waves, which are frequency independent, are also compared for theory versus experiment in Figure 10(b) on the right. Absolute time delays were calculated as phase delay in radians divided by (2*π*·frequency). The agreement between model predictions and experimental data is quite reasonable. In this sense, a system of tuned resonators with no energy transfer or connection in the axial dimension can produce experimental results heretofore interpreted as evidence of traveling waves.

## 4. Discussion

Detailed calculations show that the function of the cochlea as a real time frequency analyzer can be explained by a one-dimensional model of resonating fluid along a curved path extending from the oval window to the round window across a characteristic segment of basilar membrane, just as Helmholtz and Guyton described [14–16]. The nonlinear dependence of the resonant frequency upon the fourth power of the basilar membrane width provides for a wide dynamic range extending from tens to tens of thousands of cycles per second. Cochlear coiling is not important for frequency coding, as shown in Appendix B. However, the importance of the spiral ligament in providing a pivoting anchor point is suggested. The relatively modest effects of holes drilled into the scala tympani for experimental measurements can be modeled and indicate that such measurements are not significantly distorted by the process of observation.

In his classic treatise, *On the Sensations of Tone as a Physiological Basis for the Theory of Music [14]*, Helmholtz proposed a hypothesis of resonance, or “sympathetic vibration,” as follows: “it is probably the breadth of the membrane basilaris in the cochlea which determines tuning [as] it continually increases in width as it approaches the apex of the cochlea … then the radial fibers of the basilar membrane may be approximately regarded as forming a system of stretched strings … consequently, any exciting tone would set that part of the membrane into sympathetic vibration … that … corresponds most nearly with the exciting tone … with rapidly diminishing strength on … the adjacent parts of the membrane … Under these circumstances the parts of the membrane in unison with higher tones must be looked for near the round window, and those with the deeper, near the vertex of the cochlea [Further,] the fluid in both galleries in the cochlea must also be considered as weighting the membrane, because it cannot move without a kind of wave motion of that fluid.” Later, Guyton [38] suggested specifically that “the mass of fluid between oval and round windows and the point of vibration of the basilar membrane is the mass of the vibrating system.” These ideas together constitute a physically testable Helmholtz-Gutyon mechanism.

The present paper is dedicated to the proposition that Helmholtz and Guyton's qualitative descriptions of the essential physics of hearing were correct. However, as Helmholtz himself stated at the time “our present knowledge is not sufficient to determine with accuracy the manner in which these vibrations take place. For this purpose we require to estimate the … degree of tension and flexibility, with more precision.” New data on material properties of the basilar membrane [6, 18, 26] make a better characterized simple resonance model possible today, which includes complete specification of the relevant parameters of mass, stiffness, and damping. The present paper shows that with relatively straightforward mathematical treatment mass resonance along a curved path is sufficient to explain and predict quantitatively the major features of cochlear function. 

Although the simplicity and elegance of the resonance theory of hearing has been recognized since Helmholtz' day, twentieth century investigators felt forced to abandon this approach because of observations highly suggestive of traveling waves and large phase shifts that seemed to be impossible in a system of simple resonators akin to piano strings [22, 37, 39]. However, quantitative modeling based on modern data for the elastic moduli of the basilar membrane in both radial and axial dimensions [18] shows that apparent traveling waves and large phase shifts in response to clicks are exactly what is expected from the solution of the homogenous equation for radial bending of the basilar membrane. The latency between the onset of a punctuate acoustic stimulus at the oval window and displacement of the basilar membrane has been taken as evidence for the existence of traveling waves [10, 40]. However, as shown in Figure 10, a system of independent resonators can produce exactly this observation with zero transfer of energy in the direction of the traveling wave. In a Helmholtz-Guyton system, large apparent waves appear to travel from the characteristic place toward the apex of the cochlea. Smaller amplitude waves appear to travel from the characteristic place toward the stapes. This theoretical prediction suggests a possible unification of the resonance theory and the traveling wave theory, which have been viewed for decades as difficult to reconcile alternatives [9, 10, 22]. Traveling waves along the basilar membrane may represent emergent properties of a simple system rather than abstruse properties of a complex system.

It is not immediately obvious, however, how transient effects from the solution of the homogenous equation (10) can explain traveling waves and phase delays observed in response to continuous tones. Corresponding experimental measurements vary considerably [6]. One logical possibility is that continuous tones used as stimuli include small transients or impulses. Indeed, Bekesy's system for studying phase responses may have been quite likely to include extra signals from vibration or electrical interference from motors in the apparatus [22, 37]. This idea can be further studied in simulations such as in Figure 10, and in experiments in which various types of noise are deliberately introduced.

Further refinements of a 21st century version of resonance theory would include active components [6], nonzero resting tension in the basilar membrane associated with the spiral ligament [26], description of nonresonant motion of the basilar membrane and overtones [6], responses to complex sounds rather than pure tones, three-dimensional anatomy [3], effects of the size and design of cochleae of different animal species, and nonlinear dynamics in response to loud sounds [41, 42]. Further research may also include mechanical coupling of the inner ear model to a middle ear model, in which for example, increased fluid mass in otitis media in children or otosclerosis in older adults can be understood biomechanically. A potentially fruitful field for future biomechanical research is better characterization of viscoelastic and damping characteristics of both the ossicular ligaments and the basilar membrane.

Nevertheless, what is remarkable about Helmholtz' century old idea is how well it predicts the fundamental function of the inner ear as a real-time frequency analyzer with a wide dynamic range in terms of a set of simple second-order differential equations. Because the mathematics is straightforward, it is easy to make testable predictions and to use the model as a guide to thinking and experimentation. One hundred twenty-five years later, Helmholtz' resonance theory deserves added respect. Perhaps nature has indeed chosen a simple rather than an esoteric solution to the problem of frequency coding in the cochlea.

## Figures and Tables

**Figure 11:**
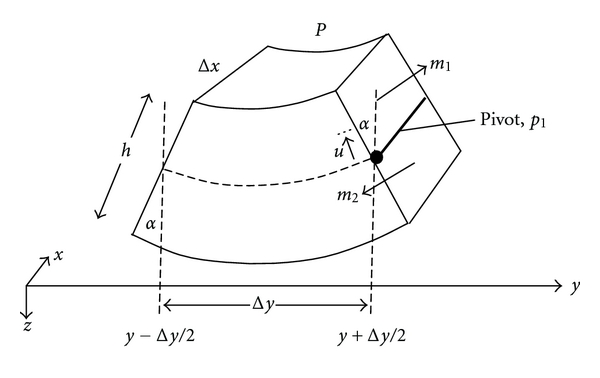


**Figure 12:**
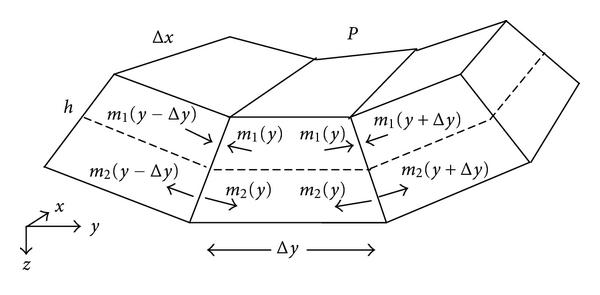


**Figure 13:**
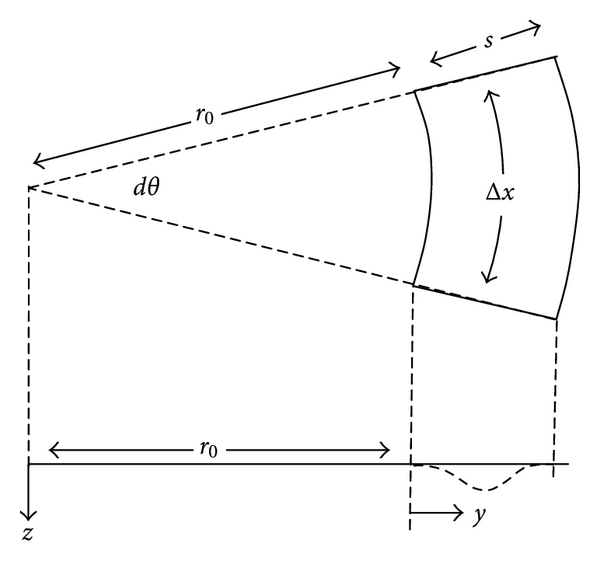


**Table 1:** Numerical estimates for model parameters.

Variable	Estimate	References
Length of basilar membrane (uncoiled)^#^	3.5 cm	Keen [23], Miller [24]
Width, *s*, of basilar membrane	0.015 cm near base to 0.056 cm near apex	Givelberg [3], Bronzino-2000, Keen [23]
Radius of scala vestibuli	0.1 cm	di Fiori [25]
Radius of scala tympani	0.15 cm	di Fiori [25]
Average cross-section of scalae	0.05 cm^2^	Liu and White [18] di Fiori [25]
Average thickness of basilar membrane	0.002 cm	Liu and White [18], Naidu and Mountain, [26], Wada et al. [27]
Viscosity of water at 37°C	0.0065 g/(cm-sec)	Lide [28]
Density of water	1.00 g/(cm)^3^	Lide [28]
Resting strain, *ε*_0_, of the basilar membrane	~ 0 (0.001)	Naidu and Mountain, [26]
Pressure gain from tympanic membrane to stapes	25	Puria et al. [29]
Damping ratio D/*E* of basilar membrane	2 × 10^−6^ sec*	Summers et al. [30], Recio et al. [31, 32], Lin Guinan [33]
Young's modulus of basilar membrane	10^9^ to 10^8^ dynes/cm^2^	Naidu and Mountain, [26] Liu and White [18]
Ratio of axial Young's modulus to radial Young's modulus of basilar membrane (axial *E*/radial *E*)	1/10	Naidu and Mountain, [26] Liu and White [18]
Linear decay formula for Young's modulus, *E *	*E* = 2 × 10^−9^ (1 − *x*/3.6)	Liu and White [18], Mammano and Nobili [5], Wada et al. [27]

^#^Anatomic dimensions in the first five rows are for human cochleae. *Computed as (*D*/*E*) = (2ln⁡⁡(2))/(*t*_1/2_ *ω* ^2^), from transient displacement of the basilar membrane in the time domain in response to clicks, where *t*_1/2_ is the half-life of the transient response and *ω* ^2^ is the characteristic angular frequency of unforced oscillation.

## Appendices

### A. Bending of a Nonrigid Elastic Membrane

It is insightful to characterize forces and moments causing membrane bending from first principles. Figure 11 shows a short segment of nonrigid elastic sheet of dimensions Δ*y* in length, *b* in width, and *h* in height, undergoing deformation by bending in the *y*-dimension in response to pressure *P* on one side. Positive deflection, *z*, is downward in Figure 11.

The dashed middle surface, which follows trajectory *z*(*y*) divides the beam into compressed (top) and stretched (bottom) halves. Consider bending moment, *m*_1_, (force *x* lever arm) about pivot, *p*_1_, produced by compression of the top half of the elastic plate during bending. There is an equal bending moment, *m*_2_, produced by stretching of the bottom half. We can find the value of *m*_1_ by integration over dimension u normal to the middle surface as the product of Young's modulus, strain, area, and lever arm

(A.1) $$\begin{array}{c} dm_{1}=E\left(\frac{u\tan\alpha }{\Delta y}\right)\left(\Delta xdu\right)u=\frac{\Delta xE\tan\alpha }{\Delta y}\cdot u^{2}du,\\ m_{1}=\frac{\Delta xE\tan\alpha }{\Delta y}\overset{\text{h}/2}{\underset{0}{\int }}u^{2}du=\frac{1}{24}\frac{\Delta xE{\text{h}}^{3}}{\Delta y}\tan\alpha . \end{array}$$

For small values of angle *α*, in the present problem, tan *α≈α*. From the Figure 11 with downward positive deflection, *z*, we have

(A.2) $$\alpha =-\frac{1}{2}\left[{\left(\frac{dz}{dy}\right)}_{y+\Delta y/2}-{\left(\frac{dz}{dy}\right)}_{y-\Delta y/2}\right]≅-\frac{\Delta y}{2}\left(\frac{d^{2}z}{dy^{2}}\right).$$

Then passing to the derivative, the bending moment associated with compression of the top half of one end of the segment at pivot point *p*_1_ is

(A.3) $$\begin{array}{cc} m_{1} & =-\frac{1}{48}\Delta xEh^{3}\left(\frac{d^{2}z}{dy^{2}}\right). \end{array}$$

Here, the second derivative term, *d* ^2^ *z*/*dy* ^2^, is negative for positive bulging of the membrane under pressure, and moment *m*_1_ is positive in sign. The goal is to characterize the forces causing vertical, *z*-directed deformation of a point on the surface *z*(*y*) of the elastic membrane in response to pressure, *P*, which may be time-varying, under either static or dynamic conditions. It is useful, therefore, to characterize moment m_1_ in terms of the *z*-directed normal force required to balance *m*_1_. Let the balancing moment be denoted *m*_1_′. Then, for variable distance, *v*, from pivot *p*_1_ along the middle surface, we have

(A.4) $$\begin{array}{cc} dm_{1}^{\prime } & =P\Delta xv\cdot dv\cdot \cos\alpha ,\\ m_{1}^{\prime } & =P\Delta x\cos\alpha \overset{\;\Delta x}{\underset{\;0}{\int }}v\;dv\\ & =\frac{1}{2}P\Delta x{\left(\Delta y\right)}^{2}\cos\alpha ≅\frac{1}{2}P\Delta x{\left(\Delta y\right)}^{2}, \end{array}$$

for small values of *α*. For balancing moments with equal absolute values *m*_1_ and *m*_1_′, we must have $\begin{array}{cc} P & =-\left(1/24\right)\left(Eh^{3}/{\left(\Delta y\right)}^{2}\right)\left(d^{2}z/{dy}^{2}\right). \end{array}$ The positive *z*-directed force created by pressure, *P*, and balancing moment m_1_ is therefore $\begin{array}{cc} P\Delta x\Delta y & =-\left(1/24\right)\left(\Delta xEh^{3}/\Delta y\right)\left(d^{2}z/{dy}^{2}\right). \end{array}$ The equal and opposite *z*-directed force created by the bending of the top half of the elastic membrane at pivot *p*_1_ and acting to balance or resist the loading pressure *P* is

(A.5) $$F_{1}=-P\Delta x\Delta y=\frac{1}{24}\frac{\Delta xEh^{3}}{\Delta y}\left(\frac{d^{2}z}{dy^{2}}\right).$$

To compute all relevant forces and moments, consider three adjacent differential volumes or nodes, each spanning Δ*y* and having thickness, *h*, and depth Δ*x*, as shown in Figure 12.

The net *z*-directed force on the center node at location *y* can be found by accounting for the vertical forces that balance moments *m*_1_(*y*) and *m*_2_(*y*) produced by compression and stretching of the top and bottom halves of the node at location y, together with the countervailing moments *m*_1_(*y* + Δ*y*) and *m*_2_(*y* + Δ*y*) produced by compression and stretching of adjacent nodes, as shown in Figure 12. The net effect of these moments in terms of the resultant *z*-directed force on the center node and the effect of pressure *P* gives a net force

(A.6) $$\begin{array}{cc} F_{net} & =P\Delta x\Delta y+\left(F_{1}\left(y\right)-F_{1}\left(y-\Delta y\right)+F_{2}\left(y\right)-F_{2}\left(y-\Delta y\right)\right)\\ & \;-\left(F_{1}\left(y\right)-F_{1}\left(y+\Delta y\right)+F_{2}\left(y\right)-F_{2}\left(y+\Delta y\right)\right). \end{array}$$

This expression can be rearranged as

(A.7) $$\begin{array}{cc} F_{net} & =P\Delta x\Delta y-\left(F_{1}\left(y+\Delta y\right)+F_{1}\left(y-\Delta y\right)-2F_{1}\left(y\right)\right)\\ & \;+\left(F_{2}\left(y+\Delta y\right)+F_{2}\left(y-\Delta y\right)-2F_{2}\left(y\right)\right)\\ & =P\Delta x\Delta y-\left(\frac{d^{2}F_{1}}{dy^{2}}\left(\Delta y^{2}\right)+\frac{d^{2}F_{2}}{dy^{2}}\left(\Delta y^{2}\right)\right).\;\; \end{array}$$

However, from the sign convention in the Figure 12 and the symmetry of the middle surface, *F*_1_ = *F*_2_, so that

(A.8) $$\begin{array}{cc} F_{net} & =P\Delta x\Delta y-2\frac{d^{2}F_{1}}{dy^{2}}\left(\Delta {\text{y}}^{2}\right)\\ & =P\Delta x\Delta y-\frac{d^{2}}{dy^{2}}\left(\frac{1}{12}\Delta x\;Eh^{3}\left(\frac{d^{2}z}{dy^{2}}\right)\right)\Delta y, \end{array}$$

where we allow in general variables *E* and *h* to be functions of *y*. In terms of the classical moment of inertia, *I* = (1/12)Δ*xh* ^3^, we have

(A.9) $$F_{net}=P\Delta x\Delta y-\frac{d^{2}}{dy^{2}}\left(EI\left(\frac{d^{2}z}{dy^{2}}\right)\right)\Delta y.$$

The concept of flexural rigidity [43] does not apply here, because the elastic membrane is not rigid. Hence, Poisson's ratio is not included in the forgoing expression. For static problems in which pressure P is constant in time, the net force is zero, and

(A.10) $$\begin{array}{c} 0=P\Delta x-\left(d^{2}/dy^{2}\right)\left(EI\left(d^{2}z/dy^{2}\right)\right)\;\text{or}\\ \left(1/12\right)\left(d^{2}/dy^{2}\right)\left(Eh^{3}\left(d^{2}z/dy^{2}\right)\right)=P. \end{array}$$

For dynamic problems, in which the membrane has local mass density, *ρ*, then *z*-directed force equals mass times acceleration, *d^2^z/dt^2^*, or

(A.11) $$F_{net}=P\Delta x\Delta y-\frac{d^{2}}{dy^{2}}\left(EI\left(\frac{d^{2}z}{dy^{2}}\right)\right)\Delta x=\rho \Delta xh\Delta y\frac{d^{2}z}{dt^{2}},$$

and so,

(A.12) $$\rho h\frac{d^{2}z}{dt^{2}}+\frac{1}{12}\frac{d^{2}}{dy^{2}}\left(Eh^{3}\left(\frac{d^{2}z}{dy^{2}}\right)\right)=P.$$

These are the fundamental differential equations for static and dynamic deformation of an elastic membrane by bending. If the membrane drives a column of fluid of total length, *u*, including membrane thickness, *h*, (Guyton's model) having mass density, *ρ*, that is, the same as that of the membrane (i.e., water density), then the static equation is unchanged, but the dynamic equation of motion in response to time-varying pressure *P*(*t*) becomes

(A.13) $$\rho u\frac{d^{2}z}{dt^{2}}+\frac{1}{12}\frac{d^{2}}{dy^{2}}\left(Eh^{3}\left(\frac{d^{2}z}{dy^{2}}\right)\right)=P\left(t\right).$$

### B. Static Deformation of the Basilar Membrane in the Radial Dimension

Consider a section of the coiled basilar membrane forming an elastic sheet of thickness, *h*, inner radius, *r*_0_, and outer radius *r*_0_ + *s*, with *s* being the span of the membrane as shown in Figure 13. The centerline arc length of the radial section is denoted Δ*x*. The radial distance from inner to outer edge of the membrane, ranging from 0 to *s*, is denoted *y*. The total radial distance from the center of curvature is *r* = *r*_0_ + *y*. The membrane is subjected to transverse pressure, *P*. The local transverse displacement of the membrane under *P* is denoted *z*. The membrane bends over surface *z*(*y*) at static equilibrium.

The fundamental equation for static bending of the membrane (Appendix A) can be written as

(B.1) $$\frac{1}{12}\frac{d^{2}}{dy^{2}}\left(E\Delta xh^{3}\left(\frac{d^{2}z}{dy^{2}}\right)\right)=P\Delta x.$$

For the radial geometry Δ*x* = *rd*,*θ* where *r* is variable and the local value of *dθ* is constant, so that

(B.2) $$Pr=\frac{h^{3}}{12}\frac{d^{2}}{dy^{2}}\left(Er\left(\frac{d^{2}z}{dy^{2}}\right)\right).$$

Let us assume that there is radial spreading of collagen fibers in the membrane, so that they attach to inner and outer supports at right angles. In this case, the local material property, Young's modulus *E*, is proportional to *n*/(*rd*)*θ*, where n is the constant number of radial collagen fibers. This means that *Er* is a constant, and in particular $Er=\overset{®}{E}\cdot \overset{®}{r}$ for the mean value of Young's modulus, $\overset{®}{E}$, measured at distance $\overset{®}{r}=r_{0}+\left(\text{s}/2\right)$ halfway between inner and outer edges of the membrane. Then, for radial fiber spreading, we have for constant *Er*

(B.3) $$Pr=\frac{\overset{®}{E}\;\overset{®}{r}\;h^{3}}{12}\frac{d^{4}z}{dy^{4}},$$

or

(B.4) $$\frac{d^{4}z}{dy^{4}}=12\frac{P}{\overset{®}{E}}\frac{r_{0}+y}{\;r_{0}+\left(\text{s}/2\right)\;\;}.$$

A polynomial solution of (B.4) can be obtained as follows. Suppose for constants, *c*,

(B.5) $$z\left(y\right)=c_{0}+c_{1}y+c_{2}y^{2}+c_{3}y^{3}+c_{4}y^{4}+c_{5}y^{5}.$$

Then using the derivatives (*dz*/*dy*) = *c*_1_ + 2*c*_2_ *y* + 3*c*_3_ *y* ^2^ + 4*c*_4_ *y* ^3^ + 5*c*_5_ *y* ^4^ through (*d* ^4^ *z*/*dy* ^4^) = 24*c*_4_ + 120*c*_5_ *y* and the boundary conditions *z*(0) = *z*(*s*) = 0 and (*dz*/*dy*)_*y*=0_ = (*dz*/*dy*)_*y*=*s*_ = 0, together with (B.4) evaluated at *y* = 0 and *y* = *s*, one can obtain six equations to solve simultaneously for constants *c*_0_ through *c*_5_. After some algebra, and taking $E=\overset{®}{E}$ for short, the result is

(B.6) $$\begin{array}{cc} z\left(y\right) & =\frac{1}{2}\frac{P}{Eh^{3}}\left(\frac{r_{0}}{r_{0}+\left(\text{s}/2\right)}\right)\\ & \;\times (s^{2}y^{2}\left(1+\frac{2}{5}\frac{s}{r_{0}}\right)2sy^{3}\left(1+\frac{3}{10}\frac{s}{r_{0}}\right)+y^{4}+\frac{1}{5}\frac{y^{5}}{r_{0}}). \end{array}$$

The boundary conditions of zero slope at *y* = 0 and *y* = *s* describe a “built-in” supports. Alternative and more anatomically realistic boundary conditions would include a built-in, bony support at the inner edge of the basilar membrane (*y* = 0) and a freely pivoting support at the outer edge (*y* = *s*) corresponding to the more flexible attachment of the spiral ligament. Then, the formal boundary conditions become *z*(0) = *z*(*s*) = 0,(*dz*/*dy*)_*y*=0_ = 0 and ((*d* ^2^ *z*)/(*dy* ^2^))_*y*=*s*_ = 0.

With these boundary conditions for a freely pivoting support at *y* = *s*, we have a similar expression

(B.7) $$\begin{array}{cc} z\left(y\right) & =\frac{1}{2}\frac{P}{Eh^{3}}\left(\frac{r_{0}}{r_{0}+\left(\text{s}/2\right)}\right)\\ & \;\times (s^{2}y^{2}\left(\frac{3}{2}+\frac{7}{10}\frac{s}{r_{0}}\right)-sy^{3}\left(\frac{5}{2}+\frac{9}{10}\frac{s}{r_{0}}\right)+y^{4}+\frac{2}{10}\frac{y^{5}}{r_{0}}). \end{array}$$

The spatial mean deflection across the span is obtained in general by integrating the polynomial in *y*,

(B.8) $$\overset{®}{z}=\frac{1}{s}\overset{\;s}{\underset{\;0}{\int }}z\left(y\right)\;dy=\frac{1}{C}\frac{Ps^{4}}{Eh^{3}},$$

where dimensionless constant, *C*, depends on the boundary conditions and also on the degree of curvature, indicated by *r*_0_. For built-in supports at both inner and outer edges in the rectangular case (*r*_0_→∞), *C* = 60. With built-in supports at both inner and outer edges in the case of maximal curvature (*r*_0_ → 0), *C* = 60 also. For a pivoting support at the outer edge in the rectangular case (*r*_0_→∞), *C* = 26.7. For a pivoting support at the outer edge in the case of maximal curvature (*r*_0_ → 0), *C* = 24. The pivoting boundary condition allows for substantially greater average deflection. The radius of curvature, *r*_0_, has minimal influence on basilar membrane deflection. (One exception for the pivoting boundary condition is a modest increase in the sensitivity of the basilar membrane to bending for tight radii of curvature versus large radii (1/24 versus 1/26.7, an 11 percent increase, other factors being equal) providing one mechanism supporting the work of Manoussaki et al. [44, 45], who describe lower frequency limits of hearing in species with tightly coiled apical cochleae.)

Knowing the mean deflection allows one to compute the “spring constant” of a segment of the basilar membrane having area *A* = *s* · Δ*x*. The force (pressure *x* area) required to bend the membrane with a mean deflection of $\overset{®}{z}$ into the required shape is

(B.9) $$PA=C\frac{AEh^{3}}{s^{4}}\overset{®}{z}.$$

The spring constant for elastic bending of a membrane bridge of area, *A*, with mean displacement, $\overset{®}{z}$, is the ratio of force to displacement, or

(B.10) $$\overset{®}{k}=C\frac{AEh^{3}}{s^{4}},$$

and the ratio

(B.11) $$\frac{\overset{®}{k}}{A}=C\frac{Eh^{3}}{s^{4}}.$$

To represent viscous damping associated with membrane bending, assume for simplicity a Kelvin-Voigt model with parallel spring *k* and damper *μ*, such that that $\overset{®}{k}=E\phi$ for shape factor, *φ*. Then, the corresponding damper for the same geometric shape is given by $\overset{®}{\mu }=D\phi =C\left(ADh^{3}/s^{4}\right)\;$ with damping modulus, D, and typically *D*/*E* ≪ 1 sec. Then, the ratio $\overset{®}{\mu }\;/A=C\left(Dh^{3}/s^{4}\right)$.

### C. Particular, Steady-State Solution for Basilar Membrane Resonance

To solve $U\ddot{x}+V\dot{x}+Wx=P_{\max}\sin\left(\omega t\right)$ using the method of undetermined coefficients, suppose that the solution *x*(*t*) and its derivatives for constants *a* and *b* are

(C.1) $$\begin{array}{cc} x\left(t\right) & =a\cdot \sin\left(\omega t\right)+b\cdot \cos\left(\omega t\right),\\ \dot{x}\left(t\right) & =a\omega \cdot \cos\left(\omega t\right)-b\omega \cdot \sin\left(\omega t\right),\\ \ddot{x}\left(t\right) & =-a\omega^{2}\cdot \sin\left(\omega t\right)-b\omega^{2}\cos\left(\omega t\right). \end{array}$$

Substituting into the characteristic equation, $U\ddot{x}+V\dot{x}+Wx-P_{\max}\sin\left(\omega t\right)=0$, we have

(C.2) $$\begin{array}{cc} & -Ua\omega^{2}\sin\left(\omega t\right)-Ub\omega^{2}\cos\left(\omega t\right)+Va\omega \cdot \cos\left(\omega t\right)\\ & \;\;-Vb\omega \text{sin}\left(\omega t\right)+Wa\sin\left(\omega t\right)+Wb\cos\left(\omega t\right)-P_{\max}\sin\left(\omega t\right)\\ & \;=0. \end{array}$$

Separating the sine and cosine terms, each of which must sum to zero for all times, *t*,

(C.3) $$\begin{array}{c} -Ua\omega^{2}-Vb\omega +Wa-P_{\max}=0,\\ -Ub\omega^{2}+Va\omega +Wb=0 \end{array}$$

so that

(C.4) $$\begin{array}{c} b=-\frac{V\omega P_{\max}}{{\left(U\omega^{2}-W\right)}^{2}+V^{2}\omega^{2}},\\ a=-\frac{\left(U\omega^{2}-W\right)P_{\max}}{{\left(U\omega^{2}-W\right)}^{2}+V^{2}\omega^{2}}. \end{array}$$

Using the phase angle transformation, $x\left(t\right)=a\sin\left(\omega t\right)+b\cos\left(\omega t\right)=\sqrt{a^{2}+b^{2}}\cdot \sin\left(\omega t+\beta \right)$, where *β* = tan^−1^ (*b/a*), we have

(C.5) $$x=\frac{P_{\max}}{\sqrt{{\left(U\omega^{2}-W\right)}^{2}+V^{2}\omega^{2}}}\text{sin}\left(\omega t+\beta \right).$$

## Nomenclature

*A*: Average cross-sectional area of scala tympani and scala vestibule
*α*: Local angle of radial bending of the basilar membrane
*b*: Length of resonant segment of coiled basilar membrane; also exponential constant in homogeneous solution of a differential equation
*β*: Phase angle between incident sound and basilar membrane displacement
*C*: Dimensionless coefficient of Eh^3^/s^4^ reflecting radial boundary conditions
*c*: Coefficients of polynomial solutions to differential equations
*D*: Damping or loss modulus of basilar membrane material
*E*: Young's modulus of basilar membrane material
*F*: Force applied to resonating fluid column in the one dimensional model
*f*: Natural frequency of sound waves
*f**: Resonant natural frequency
*h*: Local thickness of basilar membrane
*k*: Spring constant of a dx length column of elastic material, namely, *k* = *AE*/*dx*
*λ*: Constant in exponential decay of transient deformations; alsonatural angular frequency for a homogenous solution for free vibration
*m*: Mass of cochlear fluid in resonating double column; also moment of bending around a pivot point in the basilar membrane
*μ*: Damping constant of a dx length column of elastic material, namely, *μ* = *AD*/*dx*
*ω*: Angular frequency
*ω**: Resonant angular frequency
*P*: Pressure applied by the stapes to the oval window and/or by moving fluid to a patch of basilar membrane
*p*: A pivot point in the basilar membrane around which bending occurs
*φ*: Exponential function describing pressure venting by the helicotrema
*π*: Circle ratio (3.14…)
*ν*: Fluid viscosity
*r*: Mean radius of the scala tympani or scala vestibule
*ρ*: Mass density of cochlear fluid or of basilar membrane material
*s*: Width of the basilar membrane perpendicular to the spiral axis of the cochlea as a function of axial distance from the stapes
*t*: Time
*U, V, W*: Constants in a generalized second-order differential equation
*u*: Total length of a curved column of fluid extending from round to oval windows
*u, v*: Dimensions of integration of bending moments
*x*: Distance along the axis of the basilar membrane from the stapes
*y*: Radial distance from the inner bony edge of the basilar membrane to a point along the membrane
*z*: Displacement from zero pressure equilibrium of resonant segment of basilar membrane and curved, resonating fluid loop in the cochlea.
